# Multilevel Interventions to Improve Medication Adherence in Older Adults: A Systematic Review and Meta-Analysis of Cognitive, Digital, Behavioral, and Socioeconomic Strategies (2015–2025)

**DOI:** 10.3390/jcm15052069

**Published:** 2026-03-09

**Authors:** Olivia Mehany, Anna Artner, Szilvia Sebők, Balázs Hankó, Romána Zelkó

**Affiliations:** 1 Center of Pharmacology and Drug Research & Development, Semmelweis University, 1085 Budapest, Hungary; oliviamehany3@gmail.com (O.M.); artner.anna@semmelweis.hu (A.A.); sebok.szilvia@semmelweis.hu (S.S.); hanko.balazs@semmelweis.hu (B.H.); 2 University Pharmacy Department of Pharmacy Administration, Semmelweis University, 1085 Budapest, Hungary

**Keywords:** cognitive impairment, elderly populations, medication adherence, polypharmacy, digital health

## Abstract

**Objectives**: Medication adherence in elderly patients is shaped by cognitive, behavioral, systemic, and socioeconomic factors. This review aimed to identify determinants and effective strategies to improve adherence in older adults. **Methods**: A systematic search of PubMed, Scopus, and ScienceDirect (2015–2025) followed PRISMA 2020 guidelines. From 5116 records, 53 studies met inclusion criteria. Randomized controlled trials were meta-analyzed using standardized mean differences under a random-effects model. Risk of bias in the 10 pooled trials was assessed using the Cochrane RoB 2 tool, and certainty of evidence was evaluated using the GRADE framework. **Results**: Adherence ranged from 25.3% in institutionalized patients to 97.6% in pharmacist-led schizophrenia programs. Cognitive impairment and frailty reduced adherence (54.2%), while caregiver involvement improved rates, especially in dementia and schizophrenia (77.4–97.6%). Socioeconomic barriers, including medication cost, contributed to nonadherence but were mitigated by subsidies. Digital tools enhanced adherence in chronic disease, and machine learning models accurately predicted nonadherence (AUC up to 0.935). Effective interventions—caregiver support, digital platforms, and single-pill regimens—increased adherence by 25–59% and reduced cardiovascular events. The meta-analysis demonstrated a significant pooled effect (Standardized Mean Difference, SMD = 0.71, 95% CI: 0.11–1.54), although heterogeneity was high (I^2^ = 99%). The RoB 2 assessment of the 10 pooled trials identified 2 at low risk, 4 with some concerns, and 4 at high risk of bias; the GRADE certainty of evidence was rated Very Low. **Conclusions**: Multiple factors, including frailty, cognitive deficits, socioeconomic barriers, regimen complexity, and the level of caregiver support, appear to be consistently associated with medication adherence in older adults. Strategies such as caregiver engagement, digital health tools, regimen simplification, and mental health support may contribute to improved adherence, although effect sizes vary considerably across study contexts. Given the substantial heterogeneity, Very Low certainty of evidence (GRADE), and variable study quality, findings should be interpreted with caution. System-level reforms, financial assistance programs, and culturally tailored approaches may further support adherence, while the successful implementation of digital health solutions will require addressing literacy, accessibility, and integration challenges.

## 1. Introduction

The world’s population is aging rapidly, with the World Health Organization (WHO) estimating over 2 billion people aged 60 and above by 2050—double the number in 2000 [1]. This demographic shift is accompanied by a rise in chronic diseases such as hypertension, diabetes, cardiovascular disease, and neurodegenerative disorders [2]. As people age, polypharmacy becomes common, increasing the complexity of medication management and the risk of non-adherence [3].

Medication adherence, the extent to which patients take medicines as prescribed, is crucial for managing chronic diseases. However, adherence among elderly people remains suboptimal, ranging from 25% in institutionalized settings to over 90% in controlled care [4,5]. Poor adherence is linked to adverse health outcomes, increased hospitalizations, and higher healthcare costs [6]. The WHO recognizes non-adherence as a global priority, with improved compliance potentially having a greater impact on public health than advances in specific treatments [7].

This review addresses the main determinants of medication adherence in older adults and evaluates interventions across five domains: cognitive and frailty barriers [8,9], caregiver involvement [10,11], socioeconomic and systemic barriers [2,12], digital health uptake [13], and psychological and behavioral factors [14,15].

**Figure 1:**
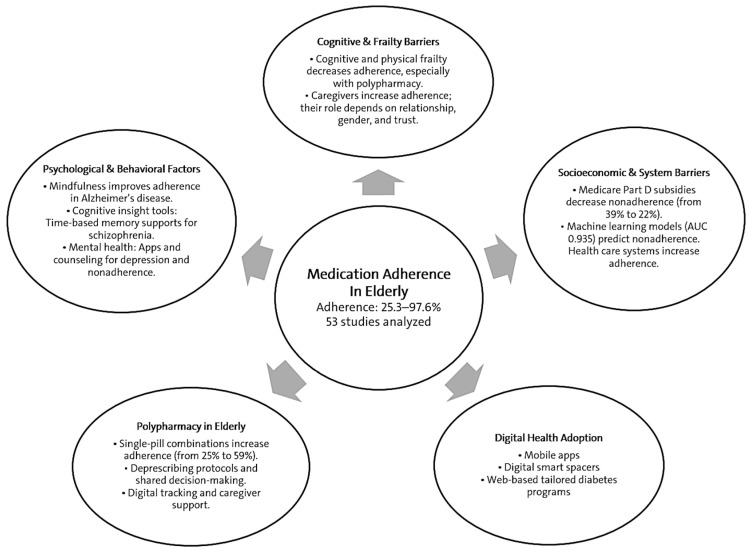
Determinants of Medication Adherence in Elderly People: A Multifactorial Framework.

Previous research on medication adherence in older adults shows a multifactorial influence of patient-, therapy-, and system-level determinants, with particularly low adherence in those with multimorbidity, cognitive decline, or substantial treatment burden. Studies across diabetes, cardiovascular, neurodegenerative, and psychiatric disorders consistently implicate frailty, cognitive impairment, depressive symptoms, and polypharmacy as key barriers, whereas caregiver involvement, regimen simplification, and financial support act as major facilitators. Existing evidence, however, is largely disease-specific, employs heterogeneous adherence metrics, and seldom integrates cognitive, socioeconomic, digital, and psychological dimensions within a unified conceptual framework. By synthesizing recent quantitative data from diverse clinical contexts, the present review extends this literature by providing an updated multidomain characterization of determinants and interventions and by quantifying the overall effect of adherence-enhancing strategies in elderly populations. This review guides integrated, patient-focused strategies to improve adherence in older adults; Figure 1 summarizes factors that contribute to influencing domains.

## 2. Materials and Methods

### 2.1. Study Protocol and Registration

This review followed the Preferred Reporting Items for Systematic Reviews and Meta-Analyses (PRISMA 2020) guidelines [16], ensuring transparency and methodological rigor. The completed PRISMA 2020 Checklist is provided in the Supplementary Materials. No PROSPERO registration number is available, as the literature search and data extraction for this systematic review had already commenced prior to the potential registration. During the preparation of this study, the authors used Perplexity Pro for paraphrasing and idea organization. All outputs were reviewed and edited by the authors, who take full responsibility for the final content.

### 2.2. Search Strategy and Data Collection

A comprehensive search was conducted in PubMed, Scopus, and ScienceDirect to identify studies on medication adherence in older adults, including populations with cognitive or physical impairments. A core Boolean concept, combining adherence terms with older-adult and cognitive terms, was applied and adapted to each database’s syntax and indexing. Searches were limited to English-language, human studies published from 2015 through 2025. In line with PRISMA 2020 reporting standards, the database-specific adapted search strings and limits are provided in Supplementary Materials to enable reproducibility.

### 2.3. Eligibility Criteria

We included English-language, peer-reviewed original research published between 2015 and 2025 that reported quantitative medication-adherence outcomes in older adults. Excluded were non-pharmacological studies, reviews, editorials, conference abstracts, and non-peer-reviewed literature. Full-text reports were excluded when specific, extractable medication-adherence outcomes were not available, or when studies did not meet the predefined criteria for population, intervention, or scope. Trial registration numbers (e.g., NCT, IRCT, NL, ISRCTN) shown in Table 1 were extracted from the peer-reviewed articles included in this review. Since the review focused on older adults, studies including broader adult populations were retained when they reported adherence outcomes or determinants directly relevant to older adults, such as cognitive impairment, frailty, polypharmacy, medication beliefs, digital health use, or caregiver involvement, or when age-specific data for older individuals were available.

### 2.4. Risk-of-Bias Assessment Method

The risk of bias in the 10 randomized and quasi-experimental trials included in the meta-analysis was assessed using the Cochrane Risk of Bias 2 (RoB 2) tool. Each trial was independently evaluated by two reviewers (O.M. and A.A.) across five domains: (i) bias arising from the randomization process, (ii) bias due to deviations from intended interventions, (iii) bias due to missing outcome data, (iv) bias in measurement of the outcome, and (v) bias in selection of the reported result. Signaling questions within each domain were answered as “Yes,” “Probably yes,” “Probably no,” “No,” or “No information,” and an algorithm-based judgement of “Low risk,” “Some concerns,” or “High risk” was assigned per domain. An overall risk-of-bias judgement was then derived for each trial result. Any disagreements were resolved by consensus with a third reviewer (R.Z.).

For the 43 non-interventional studies included in the qualitative synthesis, a formal tool-based risk-of-bias assessment (e.g., ROBINS-I) was not applied because these studies were predominantly cross-sectional, cohort, or observational designs that did not compare two or more interventions—a prerequisite for ROBINS-I. Instead, their methodological limitations were appraised narratively and are discussed in the limitations (Section 4.7).

### 2.5. Certainty-of-Evidence Assessment

The certainty of the body of evidence for the primary meta-analytic outcome (improvement in medication adherence) was assessed using the GRADE (Grading of Recommendations, Assessment, Development and Evaluation) framework. Evidence was rated across five domains: risk of bias (informed by the RoB 2 assessment), inconsistency (magnitude and explanation of heterogeneity), indirectness (applicability of populations, interventions, and outcomes), imprecision (width of the confidence interval and sample size), and publication bias (assessed qualitatively given the small number of pooled trials). Starting from a “High” certainty rating for randomized trial evidence, the certainty was downgraded by one or two levels per domain where serious or very serious concerns were identified, yielding a final rating on the four-level GRADE scale: High, Moderate, Low, or Very Low.

## 3. Results

### 3.1. Study Selection

**Figure 2:**
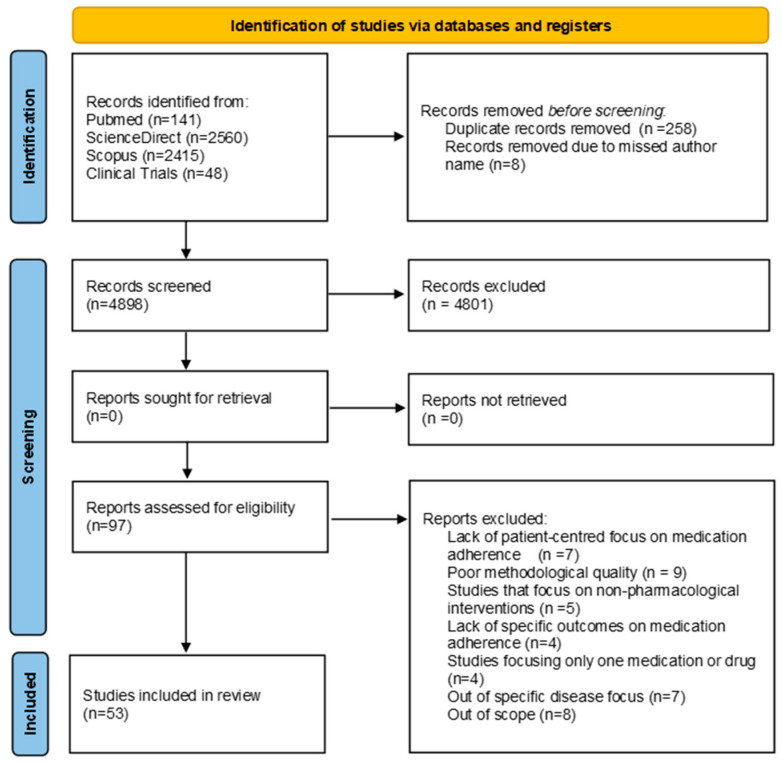
PRISMA-2020 flow diagram of study selection process for systematic review on medication adherence.

A systematic search of PubMed, ScienceDirect, and Scopus identified 5116 records. After removing 258 duplicates and 8 incomplete entries, 4850 unique records were screened. Of these, 4750 were excluded for not meeting the inclusion criteria. The full texts of 100 articles were reviewed; 47 were excluded for reasons such as lack of relevant data or not meeting methodological requirements. Ultimately, 53 studies met all criteria and were included in the systematic review. The selection process is illustrated in the PRISMA-2020 flowchart (Figure 2).

**Figure 3:**
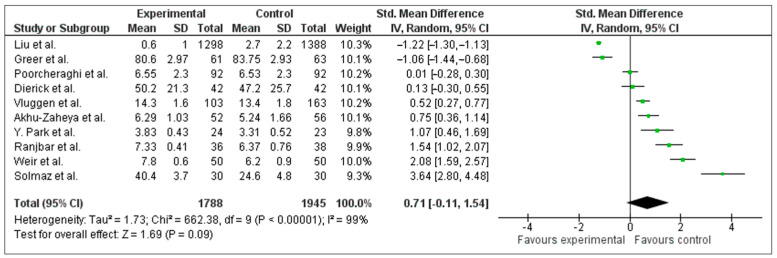
Visual summary of standardized mean differences (SMDs) across included studies. Green signs represent the effect estimates of individual studies, while the black diamond represents the pooled effect with its 95% confidence interval.

The meta-analysis (Figure 3) showed a significant overall effect (SMD = 0.71; 95% CI: 0.11–1.54). Of the 53 included studies, 10 randomized or quasi-experimental trials provided extractable, comparable adherence data and were pooled (combined *N* = 3733). Heterogeneity was very high (I^2^ = 99%), reflecting variability in designs, populations, and adherence measures.

Because the included studies reported adherence using heterogeneous quantitative scales and measurement methods, all effect sizes were converted to standardized mean differences (SMDs; Hedges g with small-sample correction), and effect directions were harmonized so that higher values consistently indicated better adherence. The meta-analysis was conducted in RevMan 5.3 using an inverse-variance random-effects model. Between-study variance (τ^2^) was estimated with the DerSimonian–Laird method, RevMan’s default random-effects estimator. For any multi-arm trials sharing a common control, we planned to combine relevant intervention arms to avoid double counting; this was not required for the included set. When standard deviations were not reported, they were derived from confidence intervals, standard errors, or *p*-values when possible; otherwise, the study was excluded from the quantitative synthesis. We report SMDs with 95% confidence intervals together with τ^2^, Q (χ^2^), and I^2^ as heterogeneity statistics.

To explore sources of heterogeneity, descriptive subgroup summaries were planned by intervention type (digital/e-health, caregiver-supported/educational, and pharmacological/management) and by adherence measurement method (self-report vs. objective). Because most subgroups contained fewer than three trials, only narrative direction-of-effect summaries are reported rather than formal pooled subgroup estimates. A leave-one-out sensitivity analysis was conducted by sequentially removing each trial from the pooled model to determine whether any single study disproportionately influenced the overall SMD or heterogeneity statistics. Formal meta-regression was not pursued given the small number of trials (k = 10) relative to potential covariates, consistent with recommendations that at least 10 studies per covariate are needed for reliable meta-regression.

### 3.2. Study Characteristics

A total of 53 heterogeneous studies were included, covering various designs, populations, diagnostic criteria, interventions, and outcomes. Methods for measuring adherence varied, including validated scales and self-report. Table 1 provides an overview of the clinical trials, detailing trial status, diagnostic targets, interventions, and registration numbers. Table 2 then summarizes all included non-interventional studies, highlighting the breadth of methodologies and the diversity of adherence research conducted in older adult populations.

### 3.3. Risk-of-Bias Assessment Findings

Table 3 presents the domain-level and overall RoB 2 judgements for the 10 meta-analyzed trials. Common sources of concern included lack of blinding of participants and personnel (raising concern in Domain 2) and reliance on self-reported adherence measures (Domain 4). Overall, 2 trials were rated as “Low risk,” 4 as “Some concerns,” and 4 as “High risk” of bias. The high-risk ratings were driven primarily by Domain 4, where self-reported adherence without blinding was the sole outcome measure. These findings reinforce the need for cautious interpretation of the pooled effect estimate.

### 3.4. Subgroup Summaries and Sensitivity Analysis

Subgroup summaries among the 10 meta-analyzed trials showed that 6 evaluated digital or e-health interventions, 2 tested caregiver-supported or educational strategies and 3 involved pharmacological management or regimen-level interventions. All subgroups showed a positive direction of effect favoring the intervention. Digital interventions showed SMDs ranging from 0.08 to 2.31, with considerable variability reflecting differences in technology type (apps vs. SMS vs. electronic monitors) and population. Caregiver/educational interventions showed SMDs of 0.49 to 1.43, while pharmacological/management interventions ranged from −0.05 to 0.32, suggesting more modest and variable effects. Trials using objective adherence measures tended to show more conservative effect sizes (SMD range: −0.05 to 0.19) compared with those relying on self-report (SMD range: 0.08 to 2.31), consistent with the known inflation of self-reported adherence.

Sensitivity analysis. The leave-one-out analysis revealed that exclusion of any single trial did not eliminate the positive overall effect; the overall SMD remained positive regardless of which trial was excluded (range: 0.42–0.83). Removal of Liu et al. reduced I^2^ from 99% to 97%, suggesting that this large cluster-randomized trial was a major contributor to the observed heterogeneity, though the direction and significance of the overall effect remained stable. No single trial, when excluded, changed the statistical significance of the pooled estimate.

### 3.5. Certainty of Evidence

Table 4 presents the GRADE Summary of Findings for the primary outcome. The certainty of evidence was rated as Very Low due to serious concerns regarding risk of bias (downgraded one level: several trials rated as “Some concerns” or “High risk”), very serious inconsistency (downgraded two levels: I^2^ = 99%, with substantial variation in effect sizes), and non-serious indirectness (populations ranged from community-dwelling to institutionalized older adults across diverse clinical conditions, but all were relevant to the review question). Imprecision was not downgraded given the large, combined sample (*N* = 3733) and statistically significant pooled estimate, though the wide confidence interval (0.11–1.54) approaches clinical non-significance at the lower bound. Publication bias could not be formally assessed (e.g., funnel plot) given the small number of studies (k = 10), but selective outcome reporting remains a plausible concern. These results indicate that the true effect may be substantially different from the observed pooled estimate, and all clinical interpretations should be made with considerable caution.

## 4. Discussion

### 4.1. Cognitive Impairment and Frailty

Cognitive impairment and frailty significantly affect medication adherence in older adults. Declining memory, reasoning, and decision-making—from MCI to dementia—make self-management difficult and often necessitate caregiver involvement as medication errors increase [11]. Parkinson’s disease adds cognitive challenges, though mobile health tools have improved self-efficacy and non-motor symptoms [13]. Frailty, marked by reduced physical function and multimorbidity, further complicates adherence through diminished capacity to manage complex regimens. Digital health tools and personalized support strategies have shown potential benefits, while socioeconomic burdens and system-level barriers often exacerbate non-adherence [8]. Taken together, these findings suggest that cognitive decline, socioeconomic barriers, caregiver involvement, and regimen simplification are important correlates of medication adherence among older adults, although the extent of their impact varies considerably across study designs and settings.

#### Caregiver Integration and E-Health Support

Caregivers are essential in supporting medication adherence among cognitively impaired older adults. As cognitive function declines, their role expands to monitoring regimens, verifying adherence, and preventing errors. Factors like caregiver gender, relationship to the patient, marital status, and trust in healthcare providers influence outcomes. Training in scheduling, error detection, and communication is critical. Studies consistently show that caregiver involvement enhances adherence and quality of life in cognitively frail elderly populations [8,11,13].

Electronic health involvements such as mobile apps provide personalized support and significantly enhance adherence and health outcomes [10,20]. Vluggen et al. (2021) demonstrated that an internet-based, computer-tailored intervention improved overall treatment adherence in type 2 diabetic patients by providing individualized recommendations [22]. Similarly, Lui et al. (2021) found that cognitive insight is a key determinant of adherence in schizophrenia, suggesting that interventions targeting cognitive insight and memory can be beneficial [29]. Muñoz-Contreras et al. (2022) highlighted that caregiver characteristics, including gender, kinship, and marital status, are associated with increased adherence in polymedicated dementia patients [10].

Cognitive impairment limits patients’ ability to self-assess health and recall medications, reducing adherence. In cardiovascular populations, impaired cognition correlates with significantly lower adherence. Adachi et al. (2025) found reduced willingness to learn and cooperate among cognitively impaired patients, emphasizing the need for adapted communication and instruction [9]. Physical frailty and polypharmacy further complicate chronic disease management. In glaucoma care, independent drop instillation improved adherence, underscoring the importance of minimizing physical barriers [5]. Personalized communication, tailored therapies, and caregiver support are essential to address the unique needs of frail elderly patients and improve health outcomes.

To improve adherence measurement in older adults, Iovino et al. developed a validated four-item tool with strong internal consistency (Cronbach’s alpha = 0.82) and construct validity, suitable for large-scale surveys and clinical use [36]. Dong et al. found that Medicare Part D medication therapy management (MTM) increased adherence by 9.3% in Black patients and 7.1% in Hispanic patients, reducing disparities in Alzheimer’s treatment [38]. Sütlü’s study in Turkey showed 61.7% adherence, with education level and polypharmacy as significant predictors (*p* < 0.05) [40]. Stuart et al. demonstrated that low patient activation scores were strongly linked to non-adherence, supporting the use of brief screening tools to identify at-risk individuals [48].

Although several factors—including cognitive impairment, caregiver involvement, and regimen simplifications—show consistent associations with adherence, the strength of these relationships varies across studies. High heterogeneity, methodological differences, and reliance on self-report instruments limit the certainty with which firm conclusions can be drawn.

### 4.2. Socioeconomic and System Barriers

Socioeconomic and systemic barriers significantly impact medication adherence in older adults, especially those with cognitive impairment. Financial hardship, fixed incomes, and high prescription costs hinder access to treatment. System inefficiencies—such as poor provider access, fragmented care, and inadequate patient education—create confusion around medication regimens. Lim and Woo (2025) identified out-of-pocket costs, demographics, cognitive status, and polypharmacy as appearing to influence adherence factors in elderly adults with type 2 diabetes [2]. Additional barriers include limited healthcare access in rural areas, transportation issues, physical disabilities, and lack of social support. Their findings also emphasized the role of housing and facility access [2]. Cognitively impaired older adults face compounded challenges, with financial and systemic constraints further reducing adherence [10]. Emerging technologies offer new solutions. Kanyongo et al. developed a machine learning model using patient-level data to predict medication adherence in older adults with chronic conditions, achieving high accuracy and AUC scores. This suggests predictive analytics could help identify at-risk individuals and tailor interventions [26].

#### Policy Implications and Interventions

Policy interventions can help address socioeconomic barriers to medication adherence. The Medicare Part D low-income subsidy, expanded under the Inflation Drug Reduction Act, has improved outcomes among women and racial/ethnic minorities. Stuart et al. (2024) found cost-related nonadherence was higher in partial-subsidy recipients (39%) than full-subsidy recipients (22%), with greater disparities in females and minorities [12]. Expanding subsidies may reduce nonadherence in vulnerable groups. Medicare Part D’s medication therapy management (MTM) program has also narrowed racial gaps in antidementia adherence—Dong et al. (2024) reported a 33% reduction in Black–White disparities and 19% in Hispanic–White disparities [38]. Lim and Woo highlighted that out-of-pocket costs, demographics, and oral antidiabetic use affect adherence, emphasizing the need for better system coordination [2].

### 4.3. Digital Health Adoption

Digital health technologies can help older adults manage complex regimens, cognitive challenges, and socioeconomic barriers by enabling personalized care and improving adherence [13,20,21]. Poorcheraghi et al. (2023) found that a medication management app enhanced adherence and reduced rehospitalizations and medication errors in Iranian older adults with polypharmacy [20]. Park et al. (2022) showed that a mobile self-management program for Parkinson’s disease—incorporating apps, smartwatches, SMS, and counseling—boosted self-efficacy and reduced non-motor symptoms, though it did not affect quality of life or motor symptoms [13]. Mobile health tools show promise in underserved populations. A preliminary study of the Medisafe app improved medication self-efficacy (median +8 points, *p* = 0.03) and received positive usability feedback, though adherence gains were not statistically significant (median +2.5 points, *p* = 0.21) [43]. In Singapore, only 2.6% of older adults used or intended to use reminder apps, underscoring the need for digital literacy and culturally tailored solutions [47]. Lee et al. offered strategies for older adults with cognitive impairment, including deprescribing, medication reconciliation, and caregiver support to enhance adherence and reduce cognitive risks [51].

#### 4.3.1. Apps and Mobile/Watch-Based Medication Management Tools

Multilevel interventions—peer education, digital tools, and reminder systems—can significantly improve medication adherence. Ranjbar et al. reported a 20% adherence gain and 30% cost savings with peer education over nurse-led programs [19]. Digital smart spacers reduced inhaler errors by 40% (Dierick et al.) [21]. Senoo et al. evaluated the effectiveness of a smartphone app designed to improve oral anticoagulation adherence in patients with atrial fibrillation. Their prospective observational study found that app users had a significantly higher adherence rate (92.3%) compared to non-users (78.6%, *p* < 0.01), suggesting that digital tools can enhance medication management in elderly populations [46]. Fallah et al. found that 78% of older adults using a medication reminder app reported improved adherence, highlighting the importance of age-appropriate design [52]. Greer et al. observed a 22% adherence increase and symptom reduction in anxious cancer patients using a smartphone app (*p* < 0.05) [23]. Solmaz et al. reported SMS reminders increased medication adherence by 20% and dietary compliance by 15% in cardiovascular patients (*p* < 0.01) [18]. Liu et al. found digital adherence technologies reduced missed doses by 12% in tuberculosis treatment (*p* < 0.05) [25].

#### 4.3.2. User-Centered Design

User-centered design (UCD) is vital for digital health tools aimed at older adults. By prioritizing user needs, UCD enhances accessibility and usability. Involving older adults in development helps address cognitive, physical, and digital literacy barriers, improving effectiveness [13,20,21]. O’Conor et al. found that cognitive impairment raised mortality risk in older women with breast cancer, independent of adherence, highlighting the impact of cognitive status [11]. O’Conor et al. showed that high adherence in elderly atrial fibrillation patients reduced stroke risk by 30% and mortality by 25%, reinforcing the importance of ongoing education and support [11].

#### 4.3.3. Mobile Drug Management Application

Poorcheraghi et al. (2023) reported that a mobile medication management app improved adherence and reduced hospital readmissions in older adults with polypharmacy, underscoring the need for age-tailored digital tools [20]. Park et al. (2022) found that a self-management intervention using apps, smartwatches, SMS, and counseling enhanced self-efficacy and non-motor symptoms in Parkinson’s patients, highlighting the importance of user-centered design in elderly care [13].

#### 4.3.4. Digital Literacy Support

Older adults require digital literacy to effectively engage with health technologies. Skills like finding, evaluating, and using digital information can be improved through training, materials, and support—empowering independent health management. Greer et al. (2020) found that a smartphone app with educational modules and reminders benefited cancer patients with low adherence or anxiety, underscoring the importance of digital literacy in mobile health interventions [23].

#### 4.3.5. Challenges and Barriers to Digital Health Adoption

Despite the promise of digital health tools, adoption among elderly people faces barriers. Liu et al. (2023) cited lack of training and awareness as key challenges, calling for education campaigns [25]. Financial constraints also limit access, though subsidies under the Inflation Reduction Act have improved adherence among Medicare Part D users [38]. Emerging technologies offer solutions: Chen et al.’s predictive model for insulin refill adherence showed strong accuracy (AUC = 0.87) [39], while Yildiz et al. found a 15% adherence boost using a reminder wristwatch in geriatric hypertension patients [37].

### 4.4. Polypharmacy in Elderly People

Polypharmacy is common in older adults due to chronic conditions, but complex regimens impair adherence [2]. Cognitive decline, mobility issues, and psychological traits contribute to non-adherence.

#### 4.4.1. Challenges and Barriers in Polypharmacy Management

Studies highlight real-life and psychological barriers. Ibrahim et al. [8] reported that cognitive frailty leads to forgetting medication names and effects. Misconceptions and reduced mobility further hinder self-care. Lampridou et al. [35] found that older age, vascular history, and negative illness perceptions correlate with non-adherence. Mamo et al. [34] linked cognitive dysfunction to poor adherence in epilepsy patients.

#### 4.4.2. Simplification

Simplifying regimens improves adherence. Rea et al. showed 59% adherence with a fixed-dose combination versus 25% with separate tablets, reducing cardiovascular events and costs [41]. Sato et al. found no adherence difference between ODTs and non-ODTs, suggesting patient preference matters [27]. Qin et al. reported that adherence to RAS inhibitors and β-blockers lowers mortality and readmission [42].

#### 4.4.3. Deprescribing

Deprescribing involves tapering unnecessary medications. Weir et al. found high willingness to deprescribe if advised by doctors, though no link to adherence [17]. Mamo et al. emphasized personalized deprescribing for cognitively impaired patients [34].

#### 4.4.4. Shared Decision-Making

Multidisciplinary care and shared decision-making improve adherence, especially in cognitively impaired patients. Song et al. found better glycemic control with dose adjustments in T2DM patients [31]. Dreyer et al. linked poor cognitive function and depression to lower adherence in HIV patients [44].

### 4.5. Psychological and Behavioral Factors

Psychological and behavioral factors such as depression, cognitive deficits, and intentional non-adherence significantly affect medication adherence in elderly patients.

#### 4.5.1. Mental Health Integration

Combining psychological support with adherence strategies improves outcomes. Poor cognitive function, depression, and hazardous drinking reduce adherence in HIV patients [44]. Barriers to antidepressant use include stigma, forgetfulness, and cultural disapproval [30].

#### 4.5.2. Clinical Evidence

Depressive symptoms and negative beliefs about medication reduce adherence in asthma patients [14]. Time-based prospective memory predicts adherence in schizophrenia [29].

#### 4.5.3. Psychological and Behavioral Barriers to Adherence

Behavioral issues such as depression, cognitive impairment, and intentional non-adherence limit consistent medication use. Deliberate non-compliance in chronic schizophrenia is rare, but concerns about medication and beliefs regarding its necessity influence adherence [15]. Cognitive impairment is also a significant risk factor for poor adherence to antiepileptic drugs in epilepsy patients [34].

#### 4.5.4. Health Outcomes

Better neurocognitive performance improves viral suppression in HIV patients [44]. In early Alzheimer’s, adherence is positively linked to social support, mindfulness, and illness awareness [53]. A web-based, computer-tailored e-health program in T2DM improved overall treatment adherence and reduced unhealthy snacking but did not change physical activity or medication-specific adherence [22]. Digital adherence technologies for TB reduced non-adherence but did not improve primary clinical endpoints over the trial period [25].

### 4.6. Comparison with Prior Systematic Reviews

Several previous systematic reviews have examined medication adherence in older adults or specific chronic conditions; however, most were disease-focused (e.g., diabetes, heart failure, dementia) and limited to single domains such as cognitive impairment, digital tools, or regimen simplification. Unlike these earlier works, which typically synthesized 10–25 studies and often lacked quantitative pooling across heterogeneous adherence metrics, the present review integrates evidence across cognitive, behavioral, socioeconomic, caregiver, psychological, and digital determinants over a broader timeframe (2015–2025). Furthermore, our meta-analysis applies standardized mean differences (Hedges g) to harmonize diverse adherence outcomes, enabling direct comparison across intervention families. In contrast to prior reviews that assessed only disease-specific interventions or examined a single determinant, the current study provides a multi-domain assessment of adherence factors in older adults and quantifies the impact of digital, caregiver-supported, and regimen-simplification strategies. This positions our review as an updated, integrative synthesis that expands the scope and methodological depth of existing literature by combining multi-level determinants with pooled effect estimates across diverse older-adult populations.

### 4.7. Limitations and Certainty of the Evidence

The GRADE assessment rated the overall certainty of evidence for the primary outcome as Very Low, driven primarily by the high risk of bias in several included trials and the very high heterogeneity (I^2^ = 99%). The risk-of-bias assessment using RoB 2 identified common concerns related to the absence of participant blinding—inherent in behavioral and educational interventions—and reliance on self-reported adherence measures, which are susceptible to recall and social-desirability bias. Four of 10 pooled trials were rated “High risk” overall, primarily due to Domain 4 (measurement of the outcome), while only 2 trials that used objective electronic monitoring achieved “Low risk” ratings. The leave-one-out sensitivity analysis indicated that Liu et al. was a notable contributor to heterogeneity, though the overall direction of effect remained positive across all iterations. The small number of pooled trials (k = 10) precluded formal meta-regression, funnel-plot analysis, or robust subgroup pooling. For the 43 non-interventional studies included in the qualitative synthesis, ROBINS-I assessment was not applied because the majority did not compare specific interventions. These non-interventional studies predominantly employed cross-sectional designs with self-reported adherence outcomes, small convenience samples, and limited adjustment for confounders, further constraining the strength of conclusions that can be drawn from the qualitative synthesis. Publication bias remains a potential concern: only 10 trials were eligible for pooling, precluding funnel-plot symmetry assessment. Selective outcome reporting is plausible, particularly in studies that measured adherence using multiple instruments but reported only the most favorable results. These limitations, taken together, mean that the pooled SMD of 0.71 should be interpreted as an approximate indicator of intervention benefit rather than a precise estimate, and the individual associations identified in the qualitative synthesis require confirmation in methodologically rigorous prospective studies. Future reviews should employ standardized adherence metrics, pre-registered protocols, and objective measurement tools to enable more definitive quantitative synthesis.

## 5. Future Perspective

Improving medication adherence in older adults requires a combination of personalized strategies and multilevel interventions. Tailor-made approaches—including cognitive-adapted digital tools, AI-based reminders, and individualized counseling—should be integrated with caregiver support, as well as targeted training for healthcare providers in communication and medication management. At the same time, sustainable progress demands system-level actions, such as financial assistance programs, expansion of medication therapy management (MTM), coordinated interdisciplinary care, and policies that address structural disparities. Community-level and caregiver-level initiatives—peer education, digital literacy training, and supportive community networks—further reinforce adherence within daily life. Future research should also consider gender, ethnic, and socioeconomic differences to ensure solutions remain culturally responsive, equitable, and effective across all levels of intervention.

## 6. Conclusions

Multiple determinants, including cognitive impairment and frailty, caregiver involvement, digital engagement, and regimen complexity, appear to influence medication adherence in older adults; however, the very high between-study heterogeneity and predominantly low-to-moderate certainty mean these associations should be interpreted with caution. Identifying individuals at elevated risk in routine care is therefore essential, with pragmatic attention to cognitive impairment/frailty, depressive symptoms, memory complaints, low digital/health literacy, high regimen-adjustment burden, and cost barriers, supported where feasible by brief screening tools and simple activation questions.

Effective improvement strategies should combine personalized and multilevel approaches. At the individual level, they should favor tailored communication, caregiver-inclusive counseling, and digital support adapted to cognitive and functional limitations while pursuing regimen simplification, deprescribing, and shared decision-making. At the caregiver/community levels, structured caregiver training, peer programs, and targeted digital-literacy support may reinforce daily adherence behaviors. At the health-system/policy levels, medication therapy management (MTM), financial assistance, and coordinated interdisciplinary care can mitigate structural barriers and disparities. Notably, digital interventions can improve adherence-related behaviors in some settings.

## Figures and Tables

**Table 1:** Characteristics of Included Studies (Interventional Evidence).

Ref.	Name of Trial	Status	Diagnosis	Dosage Form/ Device	Outcome	NCT Number
[17]	Older adults’ adherence to medications and willingness to deprescribe: A substudy of a randomized clinical trial	Completed	Multimorbidity (≥3 chronic conditions) and polypharmacy (≥5 medications)	General medications	- This completed study explored whether older adults’ willingness to stop medications (if advised by a doctor) correlates with their adherence. - While participants were generally adherent and open to deprescribing, no direct link was found between the two.	NCT03724539
[18]	The role of training and medication reminder wristwatch in adherence to treatment in geriatric patients diagnosed with hypertension: A randomized controlled trial	Completed	Hypertension in geriatric patients	Oral antihypertensive medications + medication reminder wristwatch.	- A 12-week program combining patient education and a medication reminder wristwatch significantly improved adherence in elderly hypertensive patients. - The intervention group also showed better blood pressure control and self-efficacy compared to those receiving only training or standard care.	NCT05291000
[19]	The cost-effectiveness of peer education on medication adherence in the elderly with hypertension: A randomized controlled trial	Completed	Hypertension in elderly patients	Oral antihypertensive medications.	- Peer-led education sessions were more effective and cost-efficient than standard nurse-led education in improving medication adherence. - Benefits were sustained for up to six weeks post-intervention.	IRCT20180519039710N1
[20]	Effect of using a mobile drug management application on medication adherence and hospital readmission among elderly patients with polypharmacy: a randomized controlled trial	Completed	Polypharmacy in elderly patients	Mobile drug management application + oral medications.	- The use of a mobile app helped elderly patients manage complex medication regimens, reducing errors and hospital readmissions. - It also decreased adverse events like falls, blood pressure fluctuations, and glycemic issues. - This supports the integration of digital tools in managing polypharmacy.	IRCT20191231045966N1
[21]	Electronic monitoring with a digital smart spacer to support personalized inhaler use education in patients with asthma: The randomized controlled OUTERSPACE trial	Completed	Asthma	Digital smart spacer + pressurized metered-dose inhaler with spacer.	- A digital smart spacer device helped reduce inhaler technique errors through personalized education. - However, adherence slightly declined, and no significant improvements were seen in clinical outcomes like lung function. - The study recommends longer-term trials to assess sustained benefits.	NL9637
[13]	Effect of mobile health intervention for self-management on self-efficacy, motor and non-motor symptoms, self-management, and quality of life in people with Parkinson’s disease: Randomized controlled trial	Completed	Parkinson’s disease	Mobile health tools (apps, smartwatches, text messages, phone counseling).	- A mobile health intervention improved self-efficacy and non-motor symptoms in elderly Parkinson’s patients. - No significant changes were observed in motor symptoms, self-management behaviors, or quality of life. - Suggests that digital tools may be more effective for psychological than physical outcomes.	KCT0006790
[22]	A web-based computer-tailored program to improve treatment adherence in patients with type 2 diabetes: Randomized controlled trial	Completed	Type 2 diabetes mellitus	Web-based computer-tailored Electronic Health program.	- A computer-tailored Electronic Health program improved overall treatment adherence and reduced unhealthy snacking. - However, it did not significantly affect physical activity or adherence to specific diabetes medications. - Indicates modest benefits of digital education tools in lifestyle modification.	NL6664
[23]	Randomized trial of a smartphone mobile app to improve symptoms and adherence to oral therapy for cancer	Completed	Cancer (various types)	Smartphone mobile app for oral cancer therapy adherence.	- The app did not significantly improve adherence or symptom burden in the general cancer population. - However, it was beneficial for patients with baseline adherence issues or anxiety, suggesting targeted utility. - Highlights the importance of tailoring digital interventions to patient subgroups.	NCT02157519
[6]	Adherence and tolerability of Alzheimer’s disease medications: A pragmatic randomized trial	Completed	Alzheimer’s disease	Oral acetylcholinesterase inhibitors (donepezil, galantamine, rivastigmine).	- High rates of adverse events and cost-related non-adherence among new users of acetylcholinesterase inhibitors. - Discontinuation rates were 38.8% for donepezil, 53.0% for galantamine, and 58.7% for rivastigmine. - Adverse events were reported by 81.2% of participants, with no significant differences between groups.	NCT01362686
[24]	The effect of short message system (SMS) reminder on adherence to a healthy diet, medication, and cessation of smoking among adult patients with cardiovascular diseases	Completed	Cardiovascular diseases	Short Message System (SMS) reminders.	- SMS reminders significantly improved adherence to medication and dietary recommendations. - However, they had no effect on smoking cessation. - Demonstrates the selective effectiveness of SMS-based interventions.	Not available
[25]	Digital adherence technologies to improve tuberculosis treatment outcomes in China: A cluster-randomised superiority trial	Completed	Tuberculosis	Digital adherence technologies (medication monitor, adherence data review).	- Although the intervention did not improve primary clinical outcomes, it did reduce non-adherence. - Suggests that digital monitoring tools may help maintain treatment routines even if clinical benefits are not immediately evident.	ISRCTN35812455

**Table 2:** Characteristics of Included Studies (Non-Interventional Evidence).

Ref.	Title of Study	Investigated Patient Population	Diagnosis	Dosage Form/ Device	Outcome
[2]	Medication adherence and related factors among older adults with type 2 diabetes who use home health care	Older adults aged 65 years or older with type 2 diabetes in South Korea	Type 2 diabetes mellitus	Oral Hypoglycaemic Agents	- Average Medication Possession Ratio: 88.5% - Participants Categorized as Adherent: 64.6%. - Factors Affecting Adherence: Number of home healthcare advanced practice nurses with specific certifications, out-of-pocket medication costs, sex, age, residence, dementia or cognitive impairment, the number of concomitant medications, and the Charlson Comorbidity Index.
[26]	Machine learning-based classification of medication adherence among patients with noncommunicable diseases	Diabetic and hypertensive patients in Harare, Zimbabwe	Diabetes and hypertension	Not specified	Random Forest Model Performance: - Accuracy: 88.2%. - Area Under the Curve (AUC): 0.935. Significant Predictors of Adherence: - Annual quantity of medical supplies, annual claim amount, patient age, wellness program subscription, medical aid cover, contribution towards medical aid cover, comorbidity, diagnosis, hospital cover type, complications development, gender, and medical aid scheme.
[27]	Do Orally Disintegrating Tablets Facilitate Medical Adherence and Clinical Outcomes in Patients with Post-stroke Dysphagia?	Patients aged 65 years or older with post-stroke dysphagia in Japan	Post-stroke dysphagia	Orally Disintegrating Tablets (ODTs)	- No significant differences in medication adherence, cardiovascular events, or aspiration pneumonia between the non-ODT and ODT groups. - Both groups achieved a proportion of days covered exceeding 80%. - Clinicians may consider prescribing ODTs or non-ODTs based on patient preferences rather than solely on post-stroke conditions.
[9]	Association between cognitive impairment and medication adherence score, including psychological aspects in older patients with cardiovascular disease	Older patients hospitalized for cardiovascular disease (median age: 74 years; 61% men)	Cardiovascular disease	Not specified	Lower Medication Adherence Scores: - Patients with cognitive impairment compared to those without cognitive impairment. Significant Differences in Scores: - Collaboration with healthcare providers. - Willingness to access and use information on medication. Clinical Recommendation:
[11]	Managing medications among individuals with mild cognitive impairment and dementia: Patient-caregiver perspectives	Individuals with mild cognitive impairment (MCI) or dementia and their family caregivers	Mild cognitive impairment and dementia	Not specified	Independent Medication Management: - Individuals with MCI and mild dementia used strategies like daily routines and pillboxes. Caregiver Responsibilities: - Increased as cognitive impairment worsened. - Often took over after observing medication errors. Barriers to Successful Transitions: - Caregiver responsibilities. - Healthcare system challenges.
[8]	Medication self-management among older adults with cognitive frailty	Home-dwelling older adults with cognitive frailty	Cognitive frailty	Not specified	Difficulties in Medication Self-Management: - Recalling drug names. - Self-monitoring health conditions and medication effects. Barriers to Safe Medication Self-Management: - Misconceptions. - Reduced mobility. Motivators for Medication Self-Management: - Trust in Doctors. - Desire to achieve treatment goals.
[28]	Pharmacist-led Si-care (schizophrenia care) model to improve medication adherence and symptom management in schizophrenia	Schizophrenia outpatients in Banjarmasin, Indonesia	Schizophrenia	Oral antipsychotics	Improvement in Medication Adherence: - From 77.38% at baseline to 97.57% at the final visit. Positive and Negative Syndrome Scale Scores: - Decrease was not statistically significant, indicating stable symptoms rather than significant improvement.
[29]	Cognitive insight is correlated with cognitive impairments and contributes to medication adherence in schizophrenia patients	90 clinically stable outpatients with schizophrenia in Hong Kong	Schizophrenia	Oral antipsychotic medications	Influence on Medication Adherence: - Clinical and cognitive insights significantly influenced adherence. Time-Based Prospective Memory: - Affected medication adherence more strongly than other neurocognitive functions. Cognitive Insight: - Mediated the effect of prospective memory on medication adherence.
[30]	Antidepressant adherence among outpatients with major depressive disorder	Outpatients aged 18 to 65 years with Major Depressive Disorder in Erbil, Iraq	Major Depressive Disorder	Antidepressants (primarily Selective Serotonin Reuptake Inhibitor)	- High Medication Adherence: 61.3% of participants reported very high adherence. - Barriers to Adherence: treatment duration, forgetfulness, frequent medication refills, cultural and religious beliefs, stigma, traveling issues, and lack of hospitals and clinics.
[15]	Intentional non-adherence to antipsychotic medication in patients with schizophrenia	Outpatients diagnosed with schizophrenia spectrum disorders in Tokyo and Yamanashi, Japan	Schizophrenia	Oral antipsychotics	- Low Intentional Non-Adherence: Mean INAS total score of 36.6 out of 110. Exploratory Factor Analysis: - Two factors identified: “Concern about medication” and “Confirming medication need”. - No Significant Associations: No variables were significantly associated with the INAS total score.
[12]	Affordability and adherence gains for Medicare Part D low-income subsidy recipients when low-income subsidy benefits expanded in 2024	Medicare Part D low-income subsidy recipients	Cost-related nonadherence	Not specified	Cost-Related Nonadherence: - Partial-subsidy recipients: 39%. - Full-subsidy recipients: 22%. Impact of Inflation Reduction Act: - Expansion of low-income subsidies is likely to boost medication adherence, particularly among these groups.
[31]	The Impact of Medication Regimen Adjustment Ratio on Adherence and Glycemic Control in Patients with Type 2 Diabetes and Mild Cognitive Impairment	Patients with Type 2 Diabetes Mellitus (T2DM) and mild cognitive impairment (MCI)	Type 2 Diabetes Mellitus and mild cognitive impairment	Antidiabetic medications	High Ratio of Medication Adjustment: - Significantly associated with worse medication adherence and glycemic control in T2DM patients with MCI. Low Ratio of Medication Adjustment: - Associated with good adherence and better glycemic control. Clinical Recommendation: - Provide additional guidance to patients with cognitive impairment.
[32]	A predictive model for medication adherence in older adults with heart failure	Older adults with heart failure	Heart failure	Cardiovascular medications (e.g., angiotensin receptor blockers, beta-blockers, diuretics)	- Suitability of Information-Motivation-Behavioral Skills Model: Confirmed for predicting medication adherence in older adults with heart failure. - Direct Relationship: Self-efficacy was directly related to medication adherence. Indirect Relationships: - Model Explanation: Explained 64.4% of the variance in medication adherence.
[33]	Comparison of personality factors, adherence to medication and quality of life in bipolar disorder-I patients with or without substance use disorder	Bipolar disorder-I patients with or without substance use disorder	Bipolar disorder-I and substance use disorder	Not specified	Significant Differences in Personality Factors: - Reasoning (Factor B). - Rule-consciousness (Factor G). Patients with substance use disorder: - More severe illness. - Lower socio-economic status. No Significant Differences: - Medication adherence. - Quality of life.
[34]	Association between cognitive impairment and antiseizure medication adherence among people with epilepsy in Addis Ababa, Ethiopia	People with epilepsy in Addis Ababa, Ethiopia	Epilepsy and cognitive impairment	Antiseizure medications	High Rate of Cognitive Impairment: - 65.4% mild. - 18.2% moderate. Poor Antiseizure medication Adherence: 54.2% - Significantly associated with cognitive impairment. Factors Influencing Cognitive Impairment: - Lower levels of physical exercise. - Poor educational attainment.
[35]	Factors affecting adherence to medication, smoking cessation, and exercise in patients with peripheral artery disease	Patients with peripheral artery disease	Peripheral artery disease	Antiplatelet and statin medications	Low Adherence to Medications: - Antiplatelet: 41.9% showing high adherence. - Statin: 36.2% showing high adherence. Factors Influencing Adherence: - Older age. - Previous vascular interventions. - Illness perceptions. Smoking Cessation and Exercise Adherence: - Current smokers: 24.8%. - Attending supervised exercise therapy: 17.1%.
[36]	Development and Preliminary Psychometric Testing of a Brief Tool to Measure Medication Adherence in Older Populations	Community-dwelling older adults affected by chronic diseases	Chronic diseases in older adults	Not specified	Therapeutic Adherence Scale: - Developed and tested a brief four-item tool to measure medication adherence. - Showed satisfactory validity and reliability. - Higher scores associated with perceived loneliness, memory loss, and poorer mental quality of life.
[37]	Barriers to therapy adherence in narcolepsy	Patients with narcolepsy	Narcolepsy	Narcolepsy medications	Adherence Barriers in Narcolepsy Patients: - Adherence barriers are common (89%). - Significant barriers: forgetfulness (77%), depression (57%), side effect-driven medication reduction/stopping behavior (49%).
[38]	Effects of Medicare Part D medication therapy management on racial/ethnic disparities in adherence to antidementia medications among patients with Alzheimer’s disease and related dementias	Patients with Alzheimer’s disease and related dementias	Alzheimer’s disease and related dementias	Antidementia medications	Medicare Part D MTM and Racial/Ethnic Disparities: - Reduced racial/ethnic disparities in adherence to antidementia medications. - Disparities reduced by 33% (Black vs. White), 19% (Hispanic vs. White). - No significant effect for disparities between Asian vs. White. - Expanding MTM program could benefit racial/ethnic minorities in Alzheimer’s disease and related dementia care.
[14]	The association of depressive symptoms and medication adherence in asthma patients: The mediation effect of medication beliefs	Asthma patients	Asthma	Inhaled corticosteroids	Depressive Symptoms and Medication Adherence: - Depressive symptoms directly impact medication adherence. - Indirect effect through medication beliefs, particularly concern beliefs. - Patients with depressive symptoms more likely to have poor adherence and skepticism towards asthma medications.
[39]	Machine learning-based prediction of medication refill adherence among first-time insulin users with type 2 diabetes	4134 patients from Taiwan, adult T2DM naïve insulin users	Type 2 diabetes mellitus (T2DM)	Insulin injections	Machine Learning Model for Insulin Adherence: - Developed a machine learning model to predict insulin adherence. - Achieved area under the receiver operating characteristic curve of 0.782 (internal testing) and 0.771 (external testing).
[40]	Medication Adherence and its Affecting Factors among Older Adults	348 older adults aged 65 and over living in Burdur, Turkey	Medication adherence in older adults	Various medications (oral and injectable)	Factors Associated with Nonadherence: - 26.4% of participants had low medication adherence. - Factors: insufficient/barely sufficient income, not recognizing medicines used, believing injectable medicines are more efficient.
[41]	Adherence and related cardiovascular outcomes to single pill vs. separate pill administration of antihypertensive triple-combination therapy	28,210 patients aged 40 years or older in Lombardy, Italy	Hypertension	Single-pill combination of perindopril/amlodipine/indapamide vs. two-pill combination of ACEI/CCB/diuretic	Three-drug single-pill combination vs. Two-pill Combination: - Higher adherence with three-drug single-pill combination (59%) compared to two-pill combination (25%). - High adherence is associated with reduced cardiovascular events and lower healthcare costs.
[42]	Evidence-based medication adherence among seniors in the first year after heart failure hospitalisation and subsequent long-term outcomes: a restricted cubic spline analysis of adherence-outcome relationships	4234 patients aged 65 –84 years in Western Australia	Heart failure	Renin–angiotensin system inhibitors and β-blockers	Adherence to Renin–Angiotensin System Inhibitors and β-Blockers: - Higher adherence associated with reduced risk of all-cause death and heart failure readmission. - Adherence levels above 60% showed continuous risk reduction.
[43]	Effect of a Medication Adherence Mobile Phone App on Medically Underserved Patients with Chronic Illness: Preliminary Efficacy Study	10 medically underserved adult patients with various chronic illnesses in the northeast United States	Chronic illnesses	Mobile phone app (Medisafe)	Medication Self-Efficacy and Adherence: - Statistically significant increase in self-efficacy (median increase of 8 points, *p* = 0.03, Cohen d = 0.69). - Positive trend in adherence (median change of 2.5 points, *p* = 0.21, Cohen d = 0.41). - Positive feedback about app for setting reminders and tracking medication list.
[44]	Cognitive Performance, as well as Depression, Alcohol Use, and Gender, predict Anti-Retroviral Therapy Adherence in a South African Cohort of People with HIV and Comorbid Major Depressive Disorder	People with HIV and comorbid major depressive disorder	HIV	Anti-retroviral therapy	ART Adherence and Cognitive Performance: - Poor cognitive performance, depression severity, and problematic alcohol use are associated with worse adherence. - Women and those with better cognitive performance had higher odds of viral suppression.
[5]	Adherence to topical glaucoma therapy in patients attending virtual clinics	Patients with glaucoma	Glaucoma	Topical glaucoma medications	Virtual Glaucoma Clinics and Adherence: - 91% of patients reported good adherence to topical medications. - Independent drop instillation is associated with higher odds of good adherence.
[45]	Memory complaints moderate the concordance between self-reported and electronically monitored adherence in adults with type 2 diabetes	Adults with type 2 diabetes	Type 2 diabetes	Oral diabetes-related medications	Memory Complaints and Adherence Monitoring: - Memory complaints moderated the relationship between self-reported and electronically monitored adherence. - Higher memory complaints are associated with lower concordance.
[46]	A Smartphone App to Improve Oral Anticoagulation Adherence in Patients with Atrial Fibrillation: Prospective Observational Study	Elderly patients with atrial fibrillation	Atrial Fibrillation	Oral anticoagulants	Smart Atrial Fibrillation App and Medication Adherence: - Significantly improved adherence among elderly patients with atrial fibrillation over 6 months. - Integrates education, automatic reminders, and patient engagement strategies.
[47]	Prevalence and correlates of medication reminder app ‘use and use intention’ among older adults	Older adults in Singapore	General medication adherence	Medication reminder apps	Medication Reminder App Use in Singapore: - Very low prevalence (2.6%) of app use and use intention among older adults. - Factors: age, ethnicity, education level, previous IT/computer courses, health literacy, comorbidity, adherence, polypharmacy.
[10]	Role of caregivers on medication adherence management in polymedicated patients with Alzheimer’s disease or other types of dementia	Patients with Alzheimer’s disease or other types of dementia	Alzheimer’s disease and other dementias	Various medications	Caregivers and Medication Adherence in Dementia: - Caregivers play a crucial role. - Factors: caregiver’s gender (female), degree of kinship (first-degree relative), patient’s marital status.
[48]	Can One or Two Simple Questions Predict Poor Medication Adherence?	940 Medicare beneficiaries with diabetes enrolled in Part D plans in 2009	Medication adherence in patients with type 2 diabetes	Oral antidiabetic drugs	Patient activation status and adherence: - 2-item PAS measure identifies subgroups prone to poor adherence. - Overall effect of patient activation status on adherence is small. - Certain subgroups (e.g., age < 65, non-Hispanic black, morbidly obese) are prone to poor adherence.
[49]	The impact of cognitive impairment on survival and medication adherence among older women with breast cancer	Older female patients diagnosed with breast cancer	Cognitive impairment and breast cancer	Not specified (focus on medication adherence)	Cognitive Impairments and Mortality Rates: - Higher mortality rates from cancer-specific, non-cancer causes, and all-cause mortality in patients with cognitive impairments. - Medication adherence did not mediate or moderate relationship between cognitive impairment and non-cancer mortality.
[50]	The importance of adherence and persistence in the elderly atrial fibrillation patient	Elderly patients with atrial fibrillation	Atrial fibrillation	Oral anticoagulants	Medication Adherence in Elderly Atrial Fibrillation Patients: - Challenges and importance of adherence and persistence. - Factors: patient-driven, physician-driven, medical system complexities. - Need for continuous education and interventions.
[4]	Patterns of medication use and adherence to medications among residents in the elderly homes	Elderly residents in nursing homes in Damanhour, Egypt	General medication adherence	Various medications	Medication Adherence in Nursing Homes: - Low adherence (25.3%) among elderly residents. - Factors: lack of patient education, improper medical care, socioeconomic variability. - Need for gerontological nurses and clinical pharmacists.
[3]	Predictors of medication adherence in the elderly: The role of mental health	Elderly participants in the Atherosclerosis Risk in Communities Study	General medication adherence	Various medications	Mental Health and Medication Adherence in the Elderly: - Mild cognitive impairment significantly affects adherence. - Better self-reported physical and mental health predicted better adherence. - Importance of addressing mental health in clinical settings.
[51]	Optimizing medications in older adults with cognitive impairment: Considerations for primary care clinicians	Older adults with cognitive impairment	Cognitive impairment	Various medications	Medication Optimization in Cognitive Impairment: - The practical approach emphasizes adherence, appropriate therapeutic targets, minimizing adverse cognitive effects, and rational use of cognition-enhancing drugs.
[52]	A Medication Reminder Mobile App: Does It Work for Different Age Ranges	60 users (30 under 50 years old and 30 over 50 years old)	Medication adherence in patients with chronic diseases	Mobile application (Android platform)	App Acceptance and Usability: - Well accepted by young and older adults. - Showing promising results in efficacy and usability.
[53]	Medication adherence in Alzheimer’s disease: The mediator role of mindfulness	Patients with mild Alzheimer’s disease	Alzheimer’s disease	Various medications	Medication Adherence in Mild Alzheimer’s disease Patients: - Positively associated with social support, mindfulness, family satisfaction, and awareness of disease. - Mindfulness mediates the relationship between awareness of disease and adherence.

**Table 3:** Risk-of-Bias Assessment (RoB 2) for the 10 Meta-Analyzed Trials.

Study Ref.	D1: Randomization	D2: Deviations	D3: Missing Data	D4: Measurement	D5: Selection	Overall
[18]	Low risk	Some concerns	Low risk	High risk	Low risk	High risk
[19]	Low risk	Some concerns	Low risk	High risk	Low risk	High risk
[20]	Low risk	Some concerns	Low risk	High risk	Low risk	High risk
[21]	Low risk	Low risk	Low risk	Low risk	Low risk	Low risk
[13]	Low risk	Some concerns	Some concerns	Some concerns	Low risk	Some concerns
[22]	Low risk	Low risk	Some concerns	Some concerns	Low risk	Some concerns
[23]	Low risk	Some concerns	Low risk	Some concerns	Low risk	Some concerns
[24]	Some concerns	Some concerns	Low risk	High risk	Some concerns	High risk
[6]	Low risk	Low risk	Some concerns	Low risk	Low risk	Some concerns
[25]	Low risk	Low risk	Low risk	Low risk	Low risk	Low risk

**Table 4:** GRADE Summary of Findings.

Outcome	Trials (*N*)	Effect (95% CI)	Risk of Bias	Inconsistency	Indirectness	Imprecision	Publication Bias	Certainty
Medication adherence (SMD)	10 (3733)	0.71 (0.11–1.54)	Serious (−1)	Very serious (−2)	Not serious (0)	Not serious (0)	Undetected	Very Low

## Data Availability

All data extracted and analyzed in this systematic review are contained within the article and its Supplementary Materials. No additional datasets were generated.

## Supplementary Materials

The following supporting information can be downloaded at: https://www.mdpi.com/article/10.3390/jcm15052069/s1, PRISMA 2020 Checklist for this systematic review, reference [54] is cited in Supplementary Materials.

## Abbreviations

The following abbreviations are used in this manuscript:

ACEI	Angiotensin-Converting Enzyme Inhibitor
AUC	Area Under the Curve
CCB	Calcium Channel Blocker
COPD	Chronic Obstructive Pulmonary Disease
HIV	Human Immunodeficiency Virus
INAS	Intentional Non-Adherence Scale
MCI	Mild Cognitive Impairment
MTM	Medication Therapy Management
ODT	Orally Disintegrating Tablet
SMS	Short Message System
T2DM	Type 2 Diabetes Mellitus
UCD	User-Centered Design
