# Supplementary material for: Multilevel Interventions to Improve Medication Adherence in Older Adults: A Systematic Review and Meta-Analysis of Cognitive, Digital, Behavioral, and Socioeconomic Strategies (2015–2025)
Source: J Clin Med. 2026 Mar 9;15(5):2069. doi: 10.3390/jcm15052069 (PMC12986251; doi:10.3390/jcm15052069)
Supplement: Supplementary file 1 [file jcm-15-02069-s001.zip › jcm-4184955-supplementary.pdf]

# PRISMA 2020 Checklist

| Section and Topic | Item # | Checklist item | Location where item is reported |
|---|---|---|---|
| **TITLE** | | | |
| Title | 1 | Identify the report as a systematic review. | Title page: "Multilevel Interventions to Improve Medication Adherence in Older Adults: A Systematic Review and Meta-Analysis of Cognitive, Digital, Behavioral, and Socioeconomic Strategies (2015–2025)". |
| **ABSTRACT** | | | |
| Abstract | 2 | See the PRISMA 2020 for Abstracts checklist. | Structured Abstract section (Objective, Methods, Results, Conclusion). |
| **INTRODUCTION** | | | |
| Rationale | 3 | Describe the rationale for the review in the context of existing knowledge. | Introduction, paragraphs 1–3. |
| Objectives | 4 | Provide an explicit statement of the objective(s) or question(s) the review addresses. | Abstract (Objective) and final paragraph of Introduction (paragraphs 4.) |
| **METHODS** | | | |
| Eligibility criteria | 5 | Specify the inclusion and exclusion criteria for the review and how studies were grouped for the syntheses. | Section 2.3. Eligibility Criteria. |
| Information sources | 6 | Specify all databases, registers, websites, organisations, reference lists and other sources searched or consulted to identify studies. Specify the date when each source was last searched or consulted. | Section 2.2. Search Strategy and Data Collection (PubMed, ScienceDirect, Scopus, ClinicalTrials.gov; 2015–2025). |
| Search strategy | 7 | Present the full search strategies for all databases, registers and websites, including any filters and limits used. | Section 2.2. Search Strategy and Data Collection (core search string). |
| Selection process | 8 | Specify the methods used to decide whether a study met the inclusion criteria of the review, including how many reviewers screened each record and each report retrieved, whether they worked independently, and if applicable, details of automation tools used in the process. | Sections 2.1. Study Protocol and Registration and 2.2. Search Strategy and Data Collection; Section 3.1. Study Selection and Figure 2 (PRISMA 2020 flow diagram). |
| Data collection process | 9 | Specify the methods used to collect data from reports, including how many reviewers collected data from each report, whether they worked independently, any processes for obtaining or confirming data from study investigators, and if applicable, details of automation tools used in the process. | Section 2.2. Search Strategy and Data Collection (description of data extraction feeding into Table 1, Table 2 and the meta-analysis). |
| Data items | 10a | List and define all outcomes for which data were sought. Specify whether all results that were compatible with each outcome domain in each study were sought (e.g. for all measures, time points, analyses), and if not, the methods used to decide which results to collect. | Methods text describing adherence outcomes and effect measures; Section 3.2. Study Characteristics; Table 1, Table 2; meta-analysis |

| Section and Topic | Item # | Checklist item | Location where item is reported |
|---|---|---|---|
| | | | description in Results. |
| | 10b | List and define all other variables for which data were sought (e.g. participant and intervention characteristics, funding sources). Describe any assumptions made about any missing or unclear information. | Section 3.2. Study Characteristics; Table 1 and Table 2 columns (population, diagnosis, dosage form/device, intervention type, predictors, etc.), plus related descriptions in the Results and Discussion. |
| Study risk of bias assessment | 11 | Specify the methods used to assess risk of bias in the included studies, including details of the tool(s) used, how many reviewers assessed each study and whether they worked independently, and if applicable, details of automation tools used in the process. | Section 2.4: RoB 2 applied to 10 meta-analysed trials by two independent reviewers (O.M., A.A.) with consensus adjudication (R.Z.); non-interventional studies appraised narratively. |
| Effect measures | 12 | Specify for each outcome the effect measure(s) (e.g. risk ratio, mean difference) used in the synthesis or presentation of results. | Abstract Methods and Results; meta-analysis paragraph in Section 3.1/3.2; Figure 3 (standardized mean difference, 95% CI, random-effects model). |
| Synthesis methods | 13a | Describe the processes used to decide which studies were eligible for each synthesis (e.g. tabulating the study intervention characteristics and comparing against the planned groups for each synthesis (item #5)). | Meta-analysis description in Methods (within Sections 2.2–2.3) and early Results: only randomized or quasi-experimental trials with extractable adherence data included in the quantitative synthesis. |
| | 13b | Describe any methods required to prepare the data for presentation or synthesis, such as handling of missing summary statistics, or data conversions. | Implied in meta-analysis description (conversion of adherence measures to SMD). |
| | 13c | Describe any methods used to tabulate or visually display results of individual studies and syntheses. | Section 3.2. Study Characteristics; Table 1; Table 2; Figure 3 (forest/meta-analytic plot). |
| | 13d | Describe any methods used to synthesize results and provide a rationale for the choice(s). If meta-analysis was performed, describe the model(s), method(s) to identify the presence and extent of statistical heterogeneity, and software package(s) used. | Methods (meta-analysis description in Section 2: random-effects model); Results meta-analysis paragraph and Figure 3 (SMD, 95% CI, $I^2$ = 99%). |
| | 13e | Describe any methods used to explore possible causes of heterogeneity among study results (e.g. subgroup analysis, meta-regression). | No formal subgroup/meta-regression |
| | 13f | Describe any sensitivity analyses conducted to assess robustness of the synthesized results. | |

| Section and Topic | Item # | Checklist item | Location where item is reported |
|---|---|---|---|
| | | | analyses reported. Results meta-analysis paragraph and Discussion mention extreme heterogeneity and contextual variability only. |
| Reporting bias assessment | 14 | Describe any methods used to assess risk of bias due to missing results in a synthesis (arising from reporting biases). | Publication bias could not be formally tested (funnel plot) due to small number of pooled trials (k = 10); limitation acknowledged in Discussion 4.7 |
| Certainty assessment | 15 | Describe any methods used to assess certainty (or confidence) in the body of evidence for an outcome. | Section 2.5: GRADE framework applied to primary meta-analytic outcome; Summary of Findings in Table 4 |
| **RESULTS** | | | |
| Study selection | 16a | Describe the results of the search and selection process, from the number of records identified in the search to the number of studies included in the review, ideally using a flow diagram. | Section 3.1. Study Selection; Figure 2 (PRISMA 2020 flow diagram with all counts). |
| | 16b | Cite studies that might appear to meet the inclusion criteria, but which were excluded, and explain why they were excluded. | Section 3.1. Study Selection (97 full texts screened, 44 excluded with reasons such as poor quality or lack of relevant data). |
| Study characteristics | 17 | Cite each included study and present its characteristics. | Section 3.2. Study Characteristics; Table 1 (all included studies); Table 2 (clinical trials), with full citations in the References section. |
| Risk of bias in studies | 18 | Present assessments of risk of bias for each included study. | Section 3.3: RoB 2 domain-level and overall judgements in Table 3 |
| Results of individual studies | 19 | For all outcomes, present, for each study: (a) summary statistics for each group (where appropriate) and (b) an effect estimate and its precision (e.g. confidence/credible interval), ideally using structured tables or plots. | For individual studies, summary outcome data in Table 1 and Table 2; for meta-analysed trials, effect estimates and CIs in Figure 3 and the accompanying text. |
| Results of | 20a | For each synthesis, briefly summarise the characteristics and risk of bias among contributing studies. | Results meta-analysis |

| Section and Topic | Item # | Checklist item | Location where item is reported |
|---|---|---|---|
| syntheses | 20b | Present results of all statistical syntheses conducted. If meta-analysis was done, present for each the summary estimate and its precision (e.g. confidence/credible interval) and measures of statistical heterogeneity. If comparing groups, describe the direction of the effect. | paragraph (10 randomized/quasi-experimental trials, N = 3733) and Section 3.2. Study Characteristics. Risk of bias only discussed qualitatively in the limitations. Results meta-analysis paragraph; Figure 3: SMD = 0.71, 95% CI 0.11–1.54, $I^2$ = 99%, with direction of benefit towards multilevel interventions. |
| | 20c | Present results of all investigations of possible causes of heterogeneity among study results. | No formal investigations reported; only narrative remarks on heterogeneity in Results and Discussion. |
| | 20d | Present results of all sensitivity analyses conducted to assess the robustness of the synthesized results. | |
| Reporting biases | 21 | Present assessments of risk of bias due to missing results (arising from reporting biases) for each synthesis assessed. | Formal funnel-plot analysis not feasible (k = 10); selective reporting noted as concern in Discussion 4.7 |
| Certainty of evidence | 22 | Present assessments of certainty (or confidence) in the body of evidence for each outcome assessed. | Section 3.5: GRADE certainty assessment; overall certainty rated Very Low (Table 4) |
| **DISCUSSION** | | | |
| Discussion | 23a | Provide a general interpretation of the results in the context of other evidence. | Sections 4. Discussion and 6. Conclusion (integration of determinants, interventions, and overall pooled effect with prior literature). |
| | 23b | Discuss any limitations of the evidence included in the review. | Discussion paragraphs highlighting heterogeneity, disease-specific evidence, variable adherence measures, and overall study quality; meta-analysis paragraph noting $I^2$ = 99% and limited generalizability. |
| | 23c | Discuss any limitations of the review processes used. | Discussion comments on scope (2015–2025), English-language restriction, number of meta-analyzable trials, and lack of sensitivity/subgroup analyses. |
| | 23d | Discuss implications of the results for practice, policy, and future research. | Sections 4.2. Socioeconomic |

| Section and Topic | Item # | Checklist item | Location where item is reported |
|---|---|---|---|
| | | | and System Barriers, 4.3. Digital Health Adoption, 4.4. Polypharmacy in the Elderly, 5. Future Perspective, and 6. Conclusion. |
| **OTHER INFORMATION** | | | |
| Registration and protocol | 24a | Provide registration information for the review, including register name and registration number, or state that the review was not registered. | Section 2.1. Study Protocol and Registration (PRISMA 2020 followed; no PROSPERO or other registration number → “Review not registered”). |
| | 24b | Indicate where the review protocol can be accessed, or state that a protocol was not prepared. | No protocol prepared. |
| | 24c | Describe and explain any amendments to information provided at registration or in the protocol. | Not applicable / not reported. |
| Support | 25 | Describe sources of financial or non-financial support for the review, and the role of the funders or sponsors in the review. | This research received no external funding. |
| Competing interests | 26 | Declare any competing interests of review authors. | The authors declare no conflict of interest. |
| Availability of data, code and other materials | 27 | Report which of the following are publicly available and where they can be found: template data collection forms; data extracted from included studies; data used for all analyses; analytic code; any other materials used in the review. | All data used for this study are available from the corresponding author upon request. |

*From:* Page MJ, McKenzie JE, Bossuyt PM, Boutron I, Hoffmann TC, Mulrow CD, et al. The PRISMA 2020 statement: an updated guideline for reporting systematic reviews. BMJ 2021;372:n71. doi: 10.1136/bmj.n71.
This work is licensed under CC BY 4.0. To view a copy of this license, visit https://creativecommons.org/licenses/by/4.0/
